# Are malaria elimination efforts on right track? An analysis of gains achieved and challenges ahead

**DOI:** 10.1186/s40249-019-0524-x

**Published:** 2019-02-13

**Authors:** Sunil Dhiman

**Affiliations:** Vector Management Division, Defence Research and Development Establishment, Gwalior, Madhya Pradesh 474002 India

**Keywords:** *Plasmodium*, *Anopheles*, WHO region, Malaria elimination, Antimalarial resistance, Insecticide resistance, Control programme

## Abstract

**Background:**

Malaria causes significant morbidity and mortality each year. In the past few years, the global malaria cases have been declining and many endemic countries are heading towards malaria elimination. Nevertheless, reducing the number of cases seems to be easy than sustained elimination. Therefore to achieve the objective of complete elimination and maintaining the elimination status, it is necessary to assess the gains made during the recent years.

**Main text:**

With inclining global support and World Health Organisation (WHO) efforts, the control programmes have been implemented effectively in many endemic countries. Given the aroused interest and investments into malaria elimination programmes at global level, the ambitious goal of elimination appears feasible. Sustainable interventions have played a pivotal role in malaria contraction, however drug and insecticide resistance, social, demographic, cultural and behavioural beliefs and practices, and unreformed health infrastructure could drift back the progress attained so far. Ignoring such impeding factors coupled with certain region specific factors may jeopardise our ability to abide righteous track to achieve global elimination of malaria parasite. Although support beyond the territories is important, but well managed integrated vector management approach at regional and country level using scrupulously selected area specific interventions targeting both vector and parasite along with the community involvement is necessary. A brief incline in malaria during 2016 has raised fresh perturbation on whether elimination could be achieved on time or not.

**Conclusions:**

The intervention tools available currently can most likely reduce transmission but clearing of malaria epicentres from where the disease can flare up any time, is not possible without involving local population. Nevertheless maintaining zero malaria transmission and checks on malaria import in declared malaria free countries, and further speeding up of interventions to stop transmission in elimination countries is most desirable. Strong collaboration backed by adequate political and financial support among the countries with a common objective to eliminate malaria must be on top priority. The present review attempts to assess the progress gained in malaria elimination during the past few years and highlights some issues that could be important in successful malaria elimination.

**Electronic supplementary material:**

The online version of this article (10.1186/s40249-019-0524-x) contains supplementary material, which is available to authorized users.

## Multilingual abstracts

Please see additional file 1 for translations of the abstract into the five official working languages of the United Nations.

## Background

Improved diagnosis and modern intervention tools have increased confidence in malaria elimination globally; still, the malaria parasite causes high mortality every year. This parasitic disease proclaims huge economical loss by draining considerable funds that could have been used for economic growth. It does not only cause loss of life but also interferes with the developmental achievements, weakens the culture and causes economical handicap over a long period.

Malaria is widespread in Africa and Asia, and World Health Organisation (WHO) in recent report has considered it endemic in 76 countries [1]. Nevertheless, in the recent years, there have been positive trends towards malaria shrinkage, such as, escalated investment and increase in the availability of protection tools to the needy population, particularly living in the endemic countries [2]. This happened as a result of elimination strategy that mainly emphasised on aggressive malaria control for radical elimination to shrink the disease spread in highly endemic countries by using improved and sustainable intervention tools accessible to entire at-risk population [3–8]. Massive scale up and substantial expansion of time tested interventions contributed to about 37% decline in malaria cases and 60% mortality during the last few years [1, 9, 10]. In the year 2016 alone, approximately USD 2.7 billion was invested globally in malaria elimination efforts [1], while altogether 582 million insecticide treated bed nets (ITNs) were delivered in malaria endemic countries during 2014–2016. Of these > 86% ITN distributed alone in sub-Saharan Africa region inclined the household ownership and acceptability to about 80% in 2016 as compared to 50% in 2010. This can be considered as a positive progress in line with that not only the concerted and sustainable interventions but social, demographic, cultural and behavioural beliefs, and practices also play a pivotal role in malaria contraction [11–14].

**Fig. 1:**
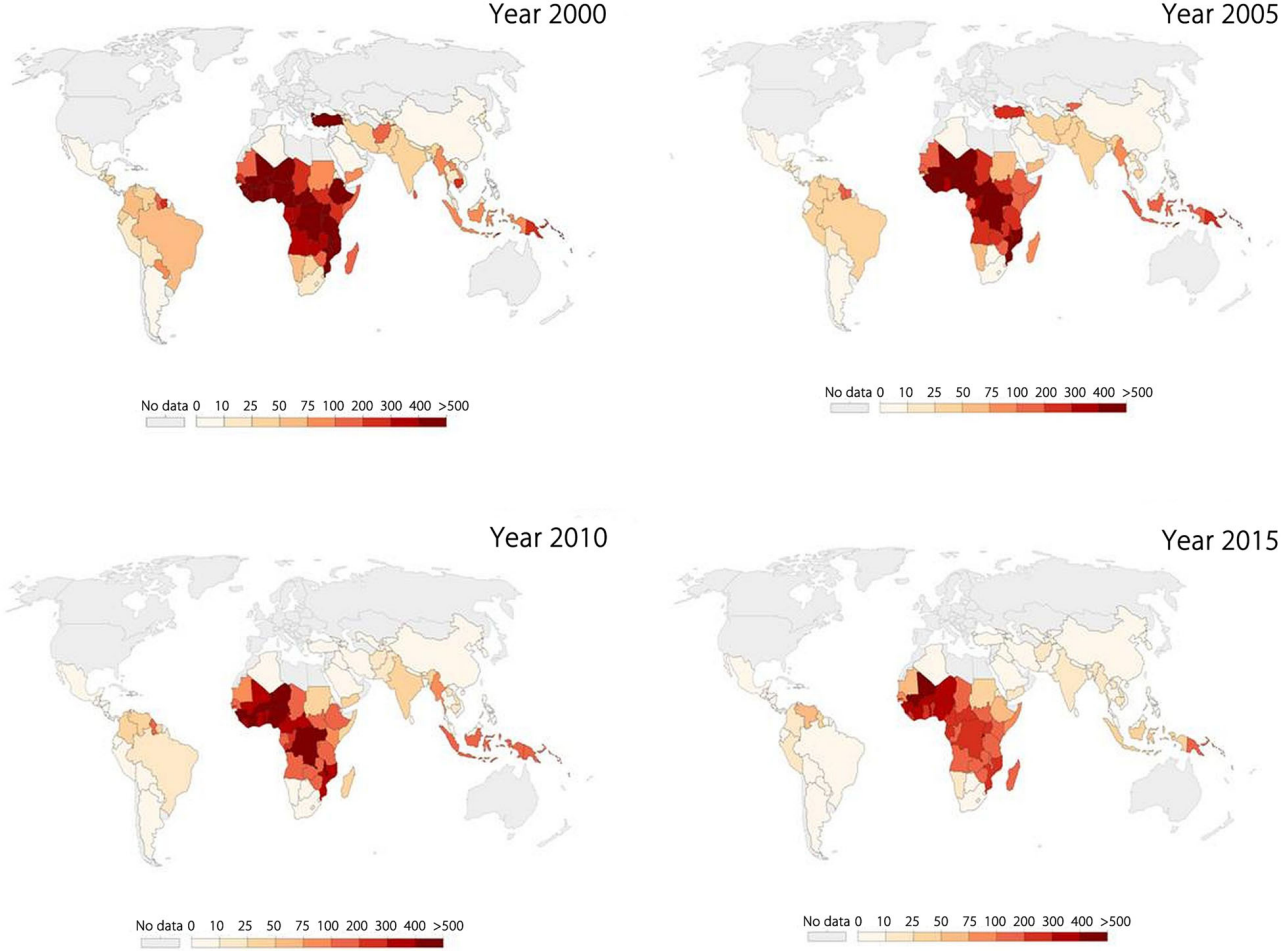
Temporal change in malaria incidences during 2000 to 2015: Country-wise change in number of malaria cases per 1000 people during the years 2000–2015 (Figure taken from www.OurWorldInData.org/malaria; assessed on 15 May 2018)

Globally malaria burden is decreasing and many countries that reported malaria previously are reporting declining trends and presumably heading towards malaria elimination (Fig. 1). Malaria control programmes have been implemented at all levels in most of the endemic countries and have yielded fruitful results by reducing overall episodes and mortality. The gains of control efforts are evident by the fact that in the year 2000, approximately 20 countries, mostly in Africa, reported an annual parasitic index of 400–500, while in the year 2015 such high burden was recorded in Mali only (Fig. 1). During the year 2016, total 44 countries reported less than 10 000 malaria cases, whereas Sri Lanka and Kyrgyzstan were addition to the certified malaria free country list of WHO. Additionally, twenty-one countries (listed as E-2020 countries by WHO) have been identified to have potential to eliminate malaria by the year 2020 on the basis of ongoing intervention activities in these countries. Nevertheless WHO is working closely to eliminate malaria from these countries, but many of them has shown increasing trends for indigenous malaria cases since 2015 [1].

Despite the progress achieved in control, malaria remains endemic in all of the WHO regions and the control gains seems to be stagnated. The achievements made so far from control towards elimination are commendable but need more focused and well planned interventions to zero down the incidences. The WHO [1, 9, 10] has charted out the global milestones for 2020 and 2025, and targets for 2030 with a vision to make world free of malaria. These milestones are based on: (I) universal access to malaria prevention, diagnosis and treatment, (II) accelerate efforts towards attainment of malaria free status and, (III) transform malaria surveillance into core intervention [1, 10], while the ultimate goal is to reduce malaria cases and deaths by 90% and to make at least 35 countries free of malaria as compared to that year 2015.

Malaria elimination is inexorable and possible also, but doubts have been raised on our ability about its complete eradication due to various reasons [15–18]. Many countries endemic today were at the verge of elimination few decades ago and it was apprehended that malaria is under control now, but it bounced back with retribution and bursted out instantly. However, recently encouraged by the progress achieved, once again elimination has gained momentum with an aim of complete eradication. Yet, there are multiple challenges which may keep on increasing as we move ahead towards complete elimination. At present, there is a pressing need to contain developing antimalarial and insecticide resistance, increase surveillance including detection of asymptomatic carriers, improve testing and treatment methods and courageous leadership at country level that could remain involved in elimination programmes and assure continuous and long-term investment. The elimination activities are primarily skewed to target *Plasmodium falciparum* and not take into account *P. vivax* which poses challenge when clearing malaria epicentres in endemic settings.

In the present review, attempt has been made to assess the progress achieved in malaria elimination in the past few years and emphasise on some issues that could be critical in successful malaria elimination globally. We have used published reports and data available online during the past few years. The literature has been searched in Scopus, PubMed and Google database using combined search strings of related key-words. Relevant data has been analysed and presented in the manuscript to reach a valid conclusion.

## Global malaria trends and prevalence in different WHO regions

The year 2016 reported approximately 216 million (95% *CI*, 196–263) confirmed malaria cases in the world. Majority of the cases were reported from WHO African Region (AFR) (90%), while South-East Asian Region (SEAR) and Eastern Mediterranean Region (EMR) contributed 7 and 2% of global malaria cases respectively [1]. Sub-Saharan Africa region has reported such a high endemicity that 15 countries in the region shared 80% of total malaria burden. Malaria incidence have been estimated to be decreased by about 11% between 2010 and 2016, but insignificant reduction in deaths to 0.445 million in 2016 as compared to 0.446 million in 2015 was reported. WHO AFR and SEAR alone contributed 91 and 6% of total deaths respectively. All the regions showed decline in malaria associated mortality in year 2016 as compared to the year 2010, but remained almost unchanged in EMR during this period.

**Fig. 2:**
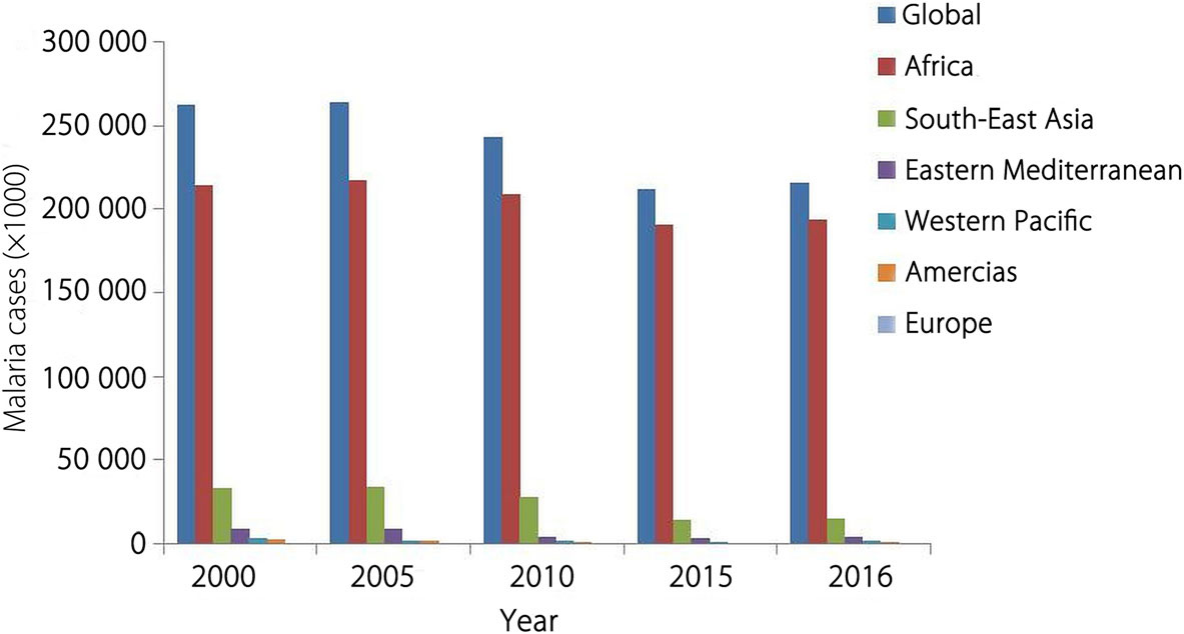
Malaria cases in the WHO regions: Malaria burden (× 1000) in WHO regions from 2000 to 2016 (Data taken from WHO, Malaria Report 2017)

**Fig. 3:**
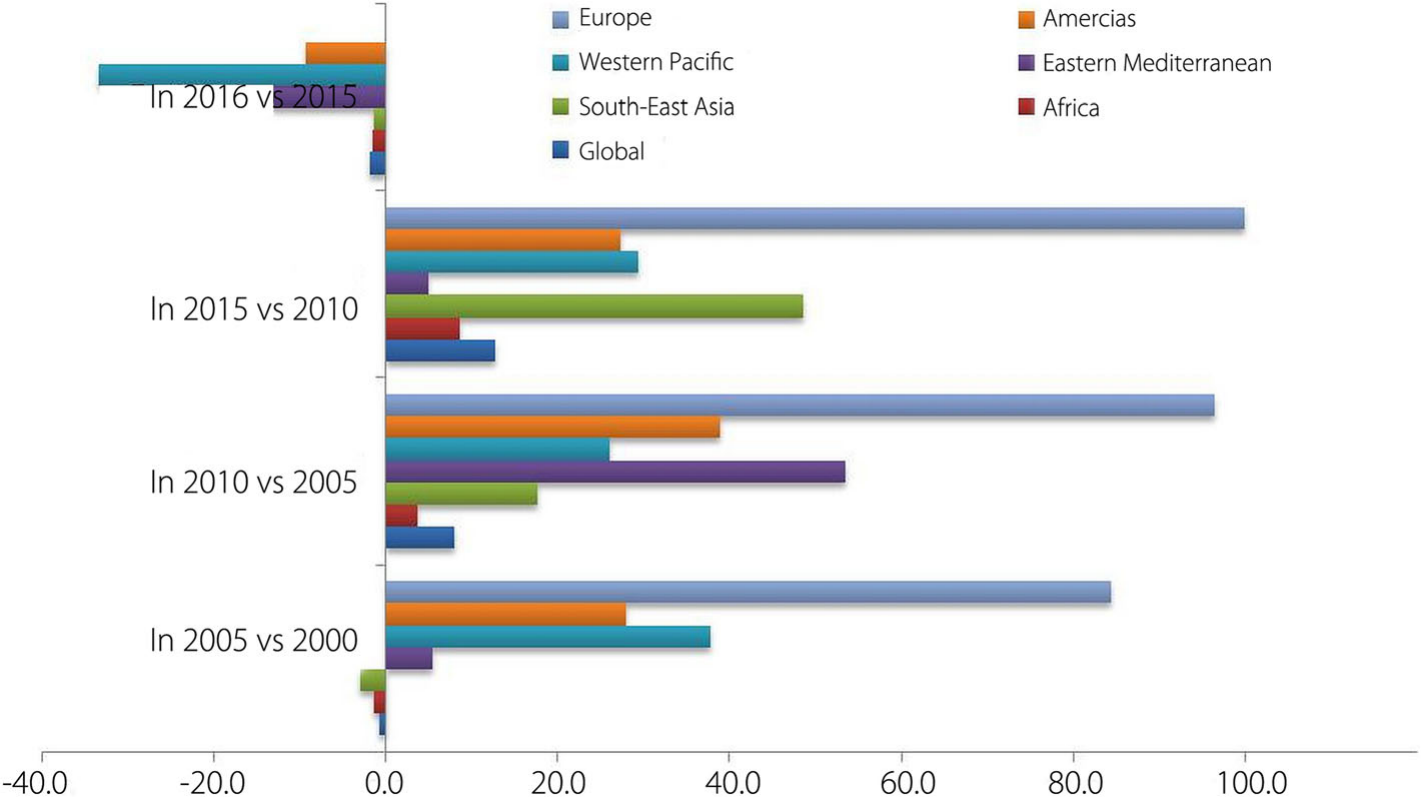
Decline in malaria cases in WHO regions: Percent (%) decrease in malaria cases from 2005 to 2016 in different WHO regions (Data taken from WHO Malaria Report 2017)

The number of confirmed malaria cases in all the six WHO regions has been shown in Fig. 2. Historically global malaria cases upsurged slightly in 2005 than in 2000, but then declined insignificantly yet steadily to the year 2015. The percent change in the confirmed cases over the years during 2000–2016 has been presented in Fig. 3. Nevertheless malaria cases again increased by about 2% in 2016 as compared to the year 2015 (Fig. 3). WHO European Region (ER) was able to reduce malaria cases virtually to zero in 2015 and 2016. However keeping that only a few cases were reported in the ER in the year 2000, the success achieved in lowering the cases to zero during 2015 and 2016, although may be important at regional level, but could not be considered breakthrough while discussing global malaria elimination.

The year 2015 has been quite fruitful for malaria shrinkage and the entire WHO regions displayed sharp decline in malaria burden. Achievements in WHO SEAR, where malaria cases were reduced by about 48% in 2015 than in 2010, were encouraging and sufficient to re-arose optimism of eliminating malaria in many endemic countries in the region. There was slight escalation in malaria cases during the year 2016 in all the WHO Regions except WHO ER. This increase in cases ranged from slightly above 1% in WHO SEAR to about 33% in WHO Western Pacific Region (WPR). Altogether this increase can be considered minimal (*P* = 0.98; *t* = 0.02) and most likely not be taken as setback to ongoing intervention programmes.

**Fig. 4:**
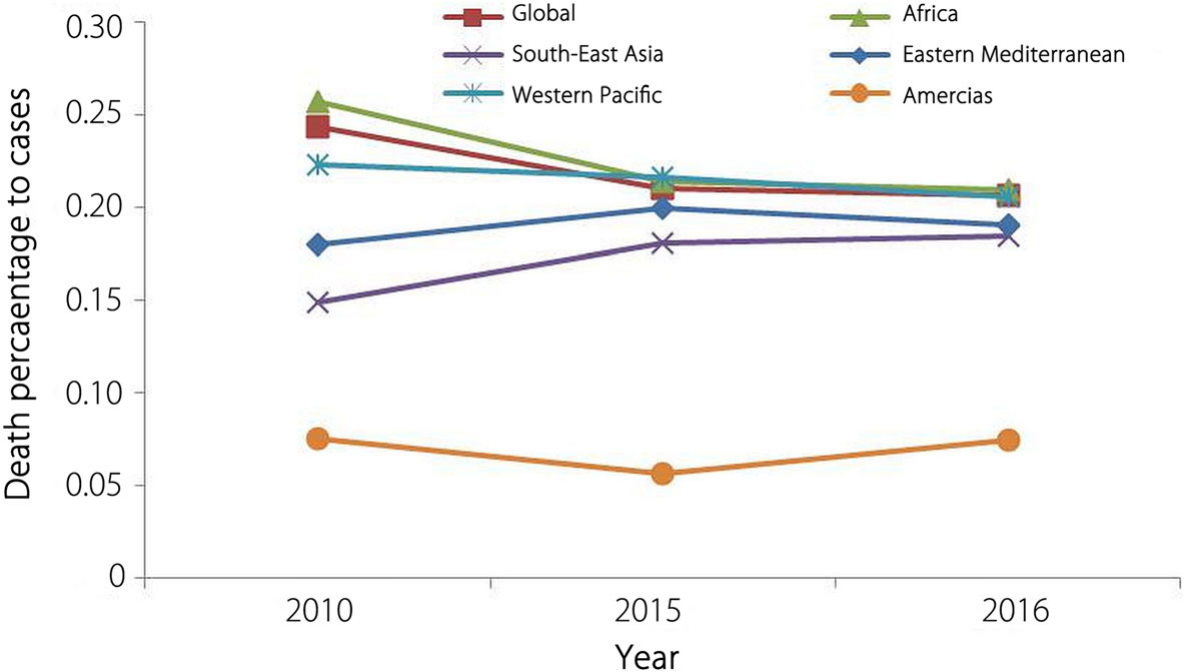
Death percentage to malaria cases: Reported death percentage to malaria cases (2010–2016) in world and WHO regions (data for European Region not included)

Malaria attributed deaths estimated in 2010 to 2016 has been presented in Table 1. Steady declining trend was recorded during 2010 (0.6 million deaths) to 2015 (0.445 million deaths), but again showed insignificant incline in 2016 (0.446 million). However statistically the change in number of estimated deaths was linear during these years and not considered significant (*F* = 0.015; *P* > 0.9). WHO AFR has shown considerable results as the death percentage to the confirmed malaria cases declined in 2015 (0.21%) than in 2010 (0.26%) and further remained stable in the year 2016 (0.21%). The progress was not much encouraging in WHO SEAR as death percentage to the malaria cases soared from 0.15% in the year 2010 to 0.18% in 2015 and 0.19% in 2016 (Fig. 4).

**Table 1:** Number of malaria deaths worldwide and WHO regions from 2010 to 2016 (Data taken from WHO Malaria Report, 2017)

WHO Region	2010	2011	2012	2013	2014	2015	2016
AFR	538 000	484 000	445 000	430 000	423 000	409 000	407 000
SEAR	41 700	34 000	29 000	22 000	25 000	26 000	27 000
EMR	7200	7100	7700	7800	7800	7600	8200
WPR	3800	3300	4000	4300	2900	2600	3300
AR	830	790	630	620	420	450	650
ER	0	0	0	0	0	0	0
Global	591 530	529 190	486 330	464 720	459 120	445 650	446 150

*AFR* African Region, *SEA* South-East Asia, *EM* Eastern Mediterranean, *WP* Western Pacific, *AR* Americas Region, *ER* European Region

Such decline in malaria burden was recorded in many WHO regions because key interventions such as ITNs coverage, rapid diagnostic tests (RDTs) were delivered in focused and systematic manner. In 2014–2016, there was substantial increase of > 50% in ITNs delivery in AFR as compared to 2011–2013. Of these, 16 most endemic countries were delivered > 80% of ITNs in AFR. Outside the AFR, majority of ITNs were delivered in endemic SEA countries during 2014–2016. Subsequently there has been increase in the household ownership of at least one ITN from 50% in 2010 to 80% in 2016. Similarly, deliveries of RDTs in AFR increased to 269 millions in 2016 than 240 millions in 2015, but in other WHO regions it decreased from 47 million in 2015 to 24 millions in 2016. Furthermore, the artemesinin-based combination therapy (ACT) treatments and preventive malaria therapy roll out in the WHO regions was also found inclined in 2016 as compared to the preceding years [1]. In contrast, the countries implementing IRS globally declined during the past few years. The major factor for lower coverage of IRS could be decline in vectors susceptibility to pyrethroids and use of other effective interventions [1, 4].

Although WHO recommended and effective interventions have been implemented to potentially accelerate malaria reduction in endemic countries, and of note, such interventions in combination have shown to reduce the malaria burden [1]. However none of the interventions can be singled out as sole driver of malaria reduction and their effectiveness may not be similar across the different geographical regions. Properly chosen interventions that could work best in a region could be critical in achieving maximum malaria reduction using control interventions [1, 2, 5].

## Challenges in achieving malaria elimination

Malaria intervention programmes were not aimed beyond control, until in 2007 when it was called for elimination by global agencies and subsequently endorsed by WHO functionaries. However opinions remained divided over such hyper optimistic objective about elimination, as diverted focus from control to elimination may change priorities around vector control, treatment and prevention [19–23]. Furthermore the aggressive efforts on elimination may not be equally fruitful in countries which at present are not fully ready to move ahead towards elimination [24–26]. While keeping cases low may comparatively be easy, but complete elimination may not be so easy in endemic countries, mainly in WHO AFR and WHO SEAR, which do not have substantial malaria free area and transmission rates have not declined considerably even after several years of continuous intervention programmes [26–29]. Reasonably, every country has peculiar and specific challenges *enroute* elimination that may include lower sensitivity to insecticides and antimalarials, cross border malaria problem, poor health system and receptivity of people to accept intervention [30–38]. Therefore understanding technical as well as operational feasibility of malaria elimination in different countries is pivotal in addition to identifying crucial challenges.

### Insecticide resistance and its impact on transmission

Although only few classes of insecticides have been approved for use in vector control programmes, but the extensive coverage with insecticidal treated nets (ITN/LLIN) and IRS in endemic areas has lowered global malaria burden during the past 15 years [1, 4, 9, 39–41]. At present, pyrethroids are the only class of insecticides used in treating bed nets to achieve protection against vector mosquitoes. WHO has documented that scale-up of ITNs has resulted to reduce malaria case incidence rates by > 50% in Africa during 2016 [1]. According to a recent report [42], approximately 663 millions of malaria cases were prevented during 2000 to 2015, of which 68% have been estimated to be averted by ITNs, whereas about 10% averted using indoor residual spray (IRS) in the endemic settings. Therefore at this crucial point of time when claims have been made to gain substantial progress in elimination by 2030, the effectiveness of insecticides needs to be maintained.

**Fig. 5:**
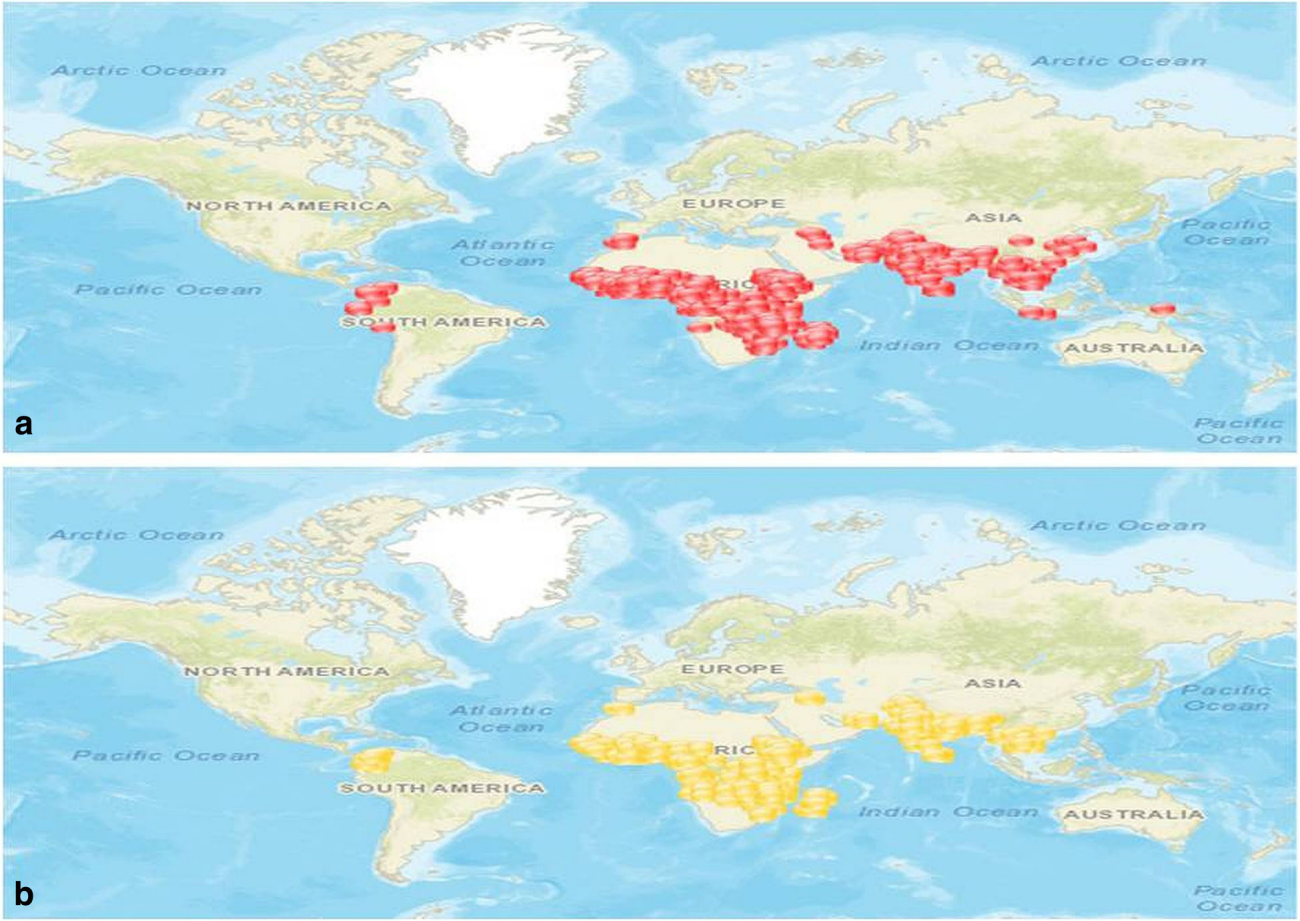
Insecticide resistance status of malaria vectors: Spatial spread of resistance against pyrethroid and/or organophosphate insecticides in major malaria vectors; (**a**-confirmed resistance marked in red; **b**-suspected resistance marked in yellow) (Figure taken from IRMapper-www.irmapper.com; assessed on 28 May 2018)

The resistance to insecticides among the *Anopheles* vectors is increasing and spreading to newer areas rapidly. Resistance in potential malaria vectors to majority of insecticides used currently has been recorded from almost entire AFR and SEAR, and gradually spreading in Americas Region (AR) also (Fig. 5a). Similarly, in many countries the susceptibility has been decreased over the years and resistance has been suspected in different malaria vectors (Fig. 5b) (For detail: visit IRMAPPER). In the Mekong region of SEAR, resistance against insecticides was reported even from several non-endemic countries (Fig. 5a, and b) which further raised concern, primarily because of the risk of vector migration to other endemic regions [1, 9]. The declining effectiveness of insecticides may have serious impact on vector control and malaria transmission. Although not investigated, but reduced sensitivity of vector mosquitoes to insecticides, in addition to other factors, could be an important factor in escalating malaria cases in 2016 as compared to 2015 [39].

Resistance to insecticides has been reported in all major anopheline vectors globally. In the current decade more than 60 countries have reported confirmed resistance to at least one class of insecticide, while about 50 of them reported resistance to at least two classes of insecticides. At present, it seems difficult to accurately estimate the extent of insecticide resistance among mosquito vectors, mainly because many endemic countries do not carry out adequate monitoring of insecticide resistance in local malaria vectors.

Certainly insecticides have been envisaged as chief drivers to reduce malaria burden in endemic areas that have susceptible vector population but their efficacy in areas with resistant *Anopheles* vectors has been debated over the years. Studies showed that implementation of ITNs use in areas reporting moderate to high resistance among the primary vectors has effectively reduced malaria incidences in many African countries [33, 40, 43, 44]. These findings suggested that efficacy of ITNs is equally maintained even in areas reporting insecticide resistance. It is most likely that such efficacy is due to physical barrier that ITNs create between human and mosquitoes. Such results could be encouraging from entomological control perspectives, but above studies were not specifically designed to evaluate the epidemiological impact of ITN use, therefore may not be sufficiently useful to draw conclusive inference. In contrast, a few studies have also argued that even low level of resistance to insecticides could increase the malaria incidences [39, 45].

The impact of ITNs, IRS and other insecticides, even if effective against vectors, may not be biologically absolute as their action relies on the biting behaviour and resting preferences of vector mosquitoes. Malaria vectors that do not bite indoors, immediately exit human houses after biting and prefer to rest on places not protected with insecticides may support malaria transmission despite scaling up interventions using insecticides. Several potential malaria vectors have been shown to display high behavioral plasticity to avoid insecticides and establishing extra-domiciliary malaria transmission [4, 5, 46]. Mosquito vectors that invade new territories, adapt to altered breeding and feeding ecology are more likely to survive in response to strengthened insecticide interventions. Studies have shown that many efficient vectors feed primarily on human to continue residual transmission but also feed enough on animals to evade the impact of insecticides that are human-targeted most of time [4, 5, 21, 43, 46]. Considering such challenges of diverse behaviours of vectors, careful mix of control technologies is important to reduce residual transmission to a level of low enough to eliminate malaria parasite.

Taken together, at present no alternative to currently used insecticide is available, the push to effectively use insecticide will largely depend upon the ability of intervention programmes to keep with the approved insecticides until more effective tools are introduced. In highly endemic countries prior to shifting focus on completely clearing malaria parasite in infected human, the elimination must rely on vector control. Hence it is predicated on ensuring that insecticide resistance be monitored and reported closely. Furthermore, in addition to filling knowledge gaps on insecticide resistance and understanding the impact of strategies followed to manage resistance, the use of new insecticides in isolation and combinations, and rotation of insecticides could be imperative and seemingly more realistic to slow down the resistance development. Indeed, chosen interventions using insecticides must rely on detailed baseline assessments of transmission and vector population attributes.

### Spreading resistance to effective antimalarial drugs

Malaria treatment using safe and effective antimalarials has been cornerstone of malaria elimination programmes. Among the human infecting *Plasmodium* species, the increasing proportion of drug resistant *P. falciparum* is alarming and imputed to rapidly emerging drug resistance in endemic countries mainly in WHO SEAR [46]. Over the past fifty years, countries in WHO SEAR have been epicentre for the evolution of resistance in malaria parasite to almost all classes of antimalarial drugs and subsequent spread to other WHO regions.

After the use of chloroquine in malaria treatment for a long time and sulfadoxine-pyrimethamine for a brief time, the artemisinin based combination therapy has been the first-line treatment for *P. falciparum* malaria in SEA countries [13, 47–50]. Although artemisinin and its derivatives are effective in isolation, but reason behind artemisinin combination therapy is to use this fast acting drug with a long acting drug to ensure that malaria parasite is completely cleared in human body. Artemisinin resistance to *P. falciparum* was first observed in Cambodia in 2008 and subsequently reported in different neighbouring countries including Laos, Myanmar, Vietnam and Thailand [51, 52]. Later on, the mutation in the *kelch 13* propeller gene which was associated with this resistance, was found spread in multiple countries including India [53–55]. A recent study has analysed the sequence data of *P. falciparum* from SEA countries and found that *kelch 13* mutations were distributed throughout the region, except Bangladesh, and increased in frequency over time during 2007–2013 [56]. This study identified 38 different *kelch 13* haplogroups scattered in SEA countries, each of which presumably representing distinct lineage of resistance. It was also suggested that resistance to forms of artemisinin combination therapy was present in the region as early as 2008 with low frequency, but rapidly increased over the time from 2008 to 2013. Study wise data from 2010 onwards on *P. falciparum* and *P. vivax* treatment outcomes for different antimalarials has been shown in Additional files 2 and 3. There has been reduced susceptibility to artemisinin based therapy in Indonesia and Thailand in WHO SEAR [1, 9, 57], however the neighbouring Cambodia has recorded a treatment failure rate of 18.2% (range: 13.8–22.6) to artesunate-amodiaquine therapy, and Lao PDR recorded a failure rate of 30.4% (range: 13.3–47.4) to dihydroartemisinin-piperaquine in WHO WPR [1, 56]. Similarly declining susceptibility to *P. vivax* even to the artemisinin based drugs in many countries in WHO SEAR and WPR has raised concern of spreading potential resistance to this parasite also.

**Fig. 6:**
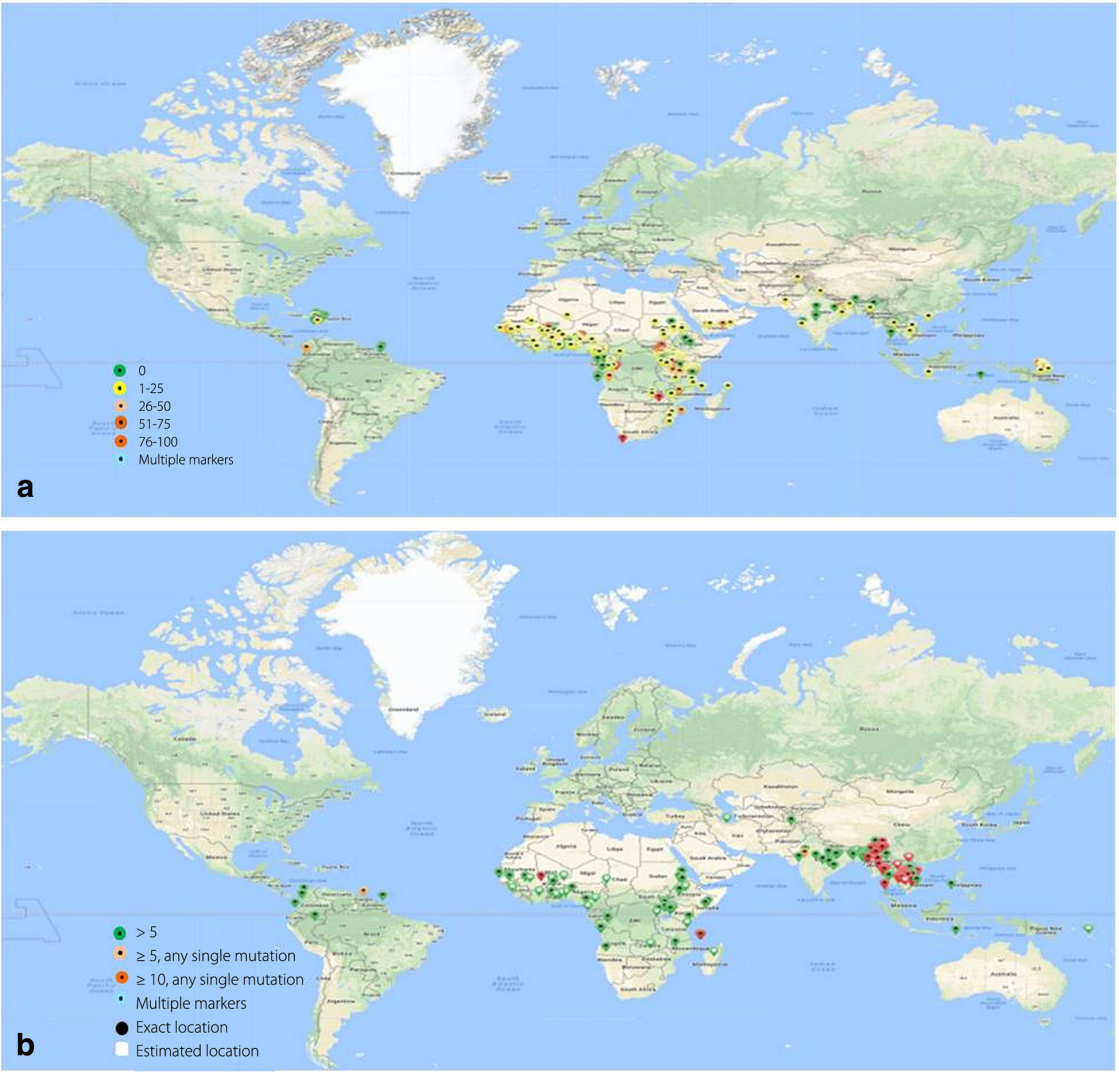
Prevalence of antimalarial resistance markers: Percent prevalence of potential antimalarial resistance markers in *P. falciparum* in different endemic countries; (**a**) *pfcrt* 76 K/T mutation for chloroquine (data from 1955 to 2018) and (**b**) *kelch 13* mutation for ACT drugs in different WHO regions. (Figure reproduced from WWARN Molecular Surveyor: assessed on 02 Jun 2018)

Artemisinin based combination therapy has begun to lose its effectiveness against *P. falciparum* in some endemic countries in WHO AFR. Studies have suggested that considerable reduction in artemisinin efficacy was recorded in some endemic countries, such as Burundi reported a treatment failure of 9.4% (median value) (range: 9.4–9.4) in the year 2015–2016. However unavailability of much data on the prevalence of artemisinin resistance in WHO AFR suggests that artemisinin based combination therapy is still effective in clearing malaria parasite. Few systematic studies in WHO AFR have indicated that *kelch 13* mutation corresponding to artemisinin is not very common [58–60], except in a few cases that displayed reduced susceptibility [61]. A recent study after performing whole genome sequence suggested that there has been emergence of indigenous artemisinin-resistant *P. falciparum* in Africa. Furthermore sequence analysis of *kelch 13* revealed a non-synonymous single nucleotide polymorphism that resulted in switching of amino acid methionine to isoleucine (M579I) [62]. The antimalarial treatment failure rates for *P. falciparum* and *P. vivax* among the patients has been presented in Additional files 2 and 3, while the prevalence of well established chloroquine resistance marker 76 K/T during 1955–2018 and established ACT resistance mutation *kelch* 13 has been shown in Fig. 6.

Although significant resources have been committed to develop more effective antimalarials during the past decades, however emergence and rapid spread of resistance has enervated the scientific efforts. The antimalarials are very effective at the beginning but subsequent clearance of parasite creates tremendous selection pressure of parasite, making it more fit to survive and proliferate even under the influence of drugs [63]. Since the appearance of antimalarials resistance on the Cambodia-Thailand border, the efforts to keep it under control has been scaled up by regular monitoring. Some initial evidences (Additional files 2 and 3) suggest that artemisinin resistance is emerging in areas adjoining Thailand and gradually spreading into Myanmar and India. This has further raised concern that resistance to artemisinin could also enter into WHO AFR via SEAR as happened previously in the context of chloroquine [1, 9, 55].

### Socio-cultural hindrance in malaria elimination

Malaria intervention efforts largely focus on the parasite as causal agent, human as host and mosquito as vector, however human behaviour, which is diverse and complex, is grossly neglected and not considered in adopting intervention strategies. This is probably because intervention experts are not well aware about how people keep with the disease and deployed control tools. An improved knowledge and understanding of community perception and beliefs about malaria as a disease and its causative agents could be useful in designing control programmes. The effective intervention tools under poor acceptability and increasing resistance are either less effective or impressive under certain conditions only [14, 64–66]. Therefore high acceptance of intervention tools which are effective to a limited extent is decisive for reigning of control programmes.

Serious malaria control efforts cannot afford to disregard human context and involvement that brace different perceptions and beliefs of malaria, malaria vectors and their management at community level [67]. Control efforts could be able to bring down malaria incidences at larger level, but may not efficiently clear disease burden at local level. This has been evident in the unprecedented decline in malaria cases in Greater Mekong Sub-Region over the past few years, but not in community populations despite the affordability to own available intervention measures [68]. Dlamini et al. [11] in a recent study conducted in Mozambique emphasised that community behaviour including delay in seeking medical attention, staying outdoors until late and maintaining stagnant water are not supportive to the national programme. Yadav et al. [14] in their study conducted in endemic north-eastern region of India reported that socio-demographic factors in remote communities significantly influence the malaria situation. Disease was more prevalent among those who have low income, poor knowledge of basic malaria facts, inhabit remote areas and hesitate to use bed nets. These communities are probably marginalised by the health assistance available in the region and have less access to ongoing control and prevention measures. Such people are not able to afford personal protection measures all the time and are vulnerable to ineffective treatment due to various cultural and financial limitations.

Gryseels et al. [68] has raised concern on achieving further reduction of malaria by using standard preventions without understanding their impact at the lowest level in the community and optimising target population involvement. Beliefs about malaria among different communities vary according to education, social, cultural and religious factors. These variations influence accepting the prevention and treatment of malaria and also activities associated with malaria control, primarily because social beliefs have no scientific and logical background, and people may have dissimilar opinions at the same time. Therefore, involving communities without conquering such beliefs acquired over the time and resolving seemingly misleading knowledge about malaria with accurate explanation is apparently difficult. Lack of awareness could also lead to serious consequences that may thwart control programmes. A recent review [69] has highlighted that ITNs distributed free of cost to the people living in endemic areas may not be used for the purpose they are meant for, but used for catching fishes in the streams and rivers and to store food material. ITNs and LLINs users are sufficiently educated about the benefits of nets while used for personal protection and also about the disadvantages when misused for deviant reasons. Nevertheless, there are preoccupied notions that traditionally popular methods used in the community are more effective and sufficient [69].

There is widespread apprehension that malaria prevalence is determined by various factors related to mosquito and parasite, but human involvement and commitment to control programmes is also important. Better understanding about the disease and interventions is crucial to overcome socio-cultural blockades. More effective health communication for elevating acceptance of prevention measures in line with control programmes is equally important. Many communities, particularly living in the far flung areas, when communicated properly and involved in anti-malaria operations may come up with novel and innovative ideas that could be useful in malaria elimination, and at a reduced financial implications. In addition to deploying effective intervention strategy, the selection of most appropriate and region or community specific tools certainly require knowledge on socio-cultural and economical aspects. Scientific understanding on factors such as movement of people and their receptivity to prevention and intervention methods affect the malaria epidemiology at local level. Oaks et al. [70] suggested that to attain effective acceptance of malaria control methods in endemic areas, control strategy must include the following considerations; (i) perception of malaria at local level, (ii) how people decide whether given treatment or preventive measures are efficacious, (iii) treatment seeking pattern of community, and (iv) involvement of communities in overall planning and evaluation of control programs.

Malaria elimination efforts require long term goals of sustainability while acknowledging social-cultural elements. Control programmes need to be designed and implemented for effectiveness and sustainability without ignoring social beliefs, perceptions, cultural and other associated priorities of the communities. Proper understanding of inter-relation between deployable intervention measures and perceived obstacles in the target population is important in long term sustainability. Community support in elimination programmes could only be ensured when people are provided culturally appropriate awareness about malaria, consequences of epidemics and difficulties in elimination. Large scale control programmes must account social contexts and cultural practices of the communities to enhance the effectiveness of the programmes.

### *Plasmodium vivax*: Challenge in elimination

**Fig. 7:**
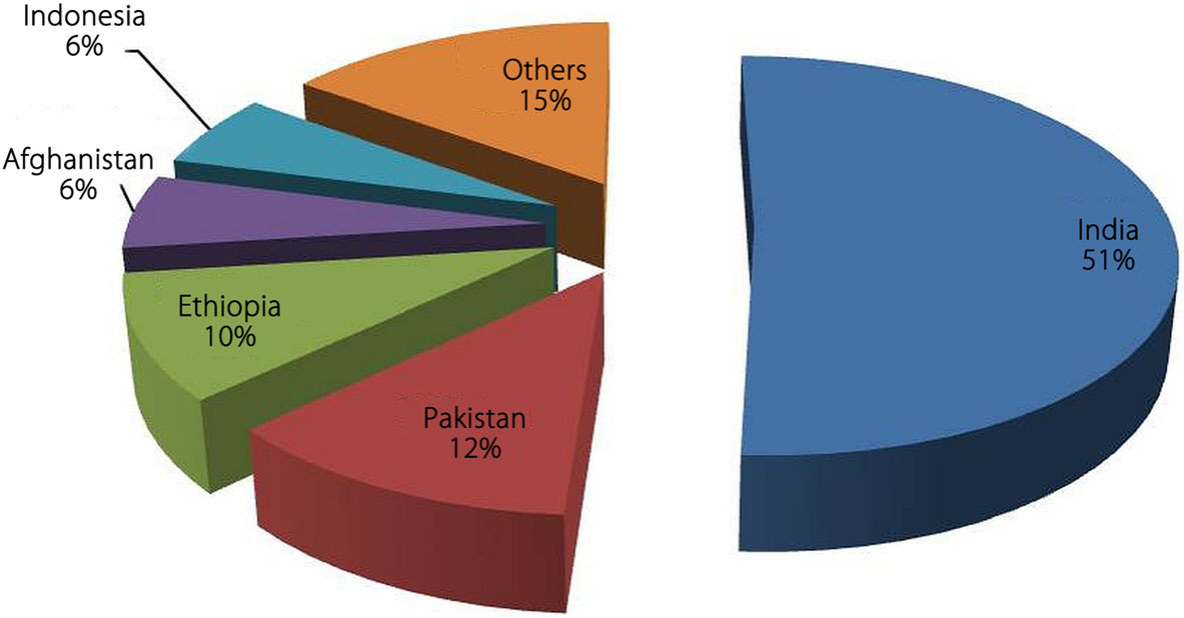
*Plasmodium vivax* burden in some endemic countries: Estimated *P. vivax* cases in five most vivax malaria endemic countries in the world during the year 2016 (Data taken from WHO Malaria Report, 2017)

*P. vivax* is not a much studied malaria parasite, probably because it is not responsible for amount of mortality and morbidity that could be compared to *P. falciparum*. Although *P. falciparum* is the most prevalent malaria parasite globally (about 95% cases during 2016), but *P. vivax* predominates in WHO AR (approximately 64% cases), SEAR (> 30% cases) and EMR (about 40% cases). In the year 2016, about 85% of estimated *P. vivax* cases were reported from the five countries namely Afghanistan, Ethiopia, India, Indonesia and Pakistan (Fig. 7) [1, 9].

Historically priority has been limited on eliminating *P. falciparum* malaria as it is more pathogenic. During the past few decades many endemic countries have reported virtually zero incidence of malaria, however to achieve complete elimination, it is obligatory to focus on tackling vivax malaria [1, 48, 49, 71, 72] In fact, control of vivax malaria is rather difficult due to the development of gametocytes within few days of infection even before patient decide to take treatment [46]. Therefore most of time human host may have sufficient parasite load to continue transmission even before the diagnosis. Several distinct biological characteristics of *P. vivax* affect its distribution and pose challenges for its elimination. *P. vivax* has comparatively broader geographical range, develops at wider temperature scale, and has longer transmission period [50, 72, 73]. Furthermore, ITNs and IRS intended to protect human may not always be equally effective to minimise *P. vivax* transmission, as some mosquito vectors in vivax endemic regions have early biting, outdoor feeding and outdoor resting behaviours [74].

Nevertheless, the most worrisome feature of *P. vivax* is the formation of dominant hypnozoite stage, hence the disease reservoir may always be present in the population even after repeated negative diagnosis. It has been shown that different strains of *P. vivax* have different latency periods which startle the activity and efficacy of available schizontocidal drugs and radical therapy. *P. vivax* infections generally appears in low parasitaemia and many time as mixed infection along with *P. falciparum,* and transmitted together [75–77], therefore more likely to be missed in routine microscopy and rapid diagnostic tests which are primarily aimed at treatment of patients. This leads to under estimation of the prevalence of infections particularly in region having low transmission and embarking on elimination.

Currently primaquine is the only anti-relapse drug available, but it may not always be recommended to produce desired results in radical cure due to various reasons. This drug is primarily effective against *P. vivax* hypnozoites, but induces haemolysis in patients with G6PD enzyme deficiency. Patients with hereditary Cyt-P450 polymorphism (CYP2D6) may have reduced primaquine bioavailability because of the interaction between CYP2D6 and primaquine metabolism [78, 79]. Therefore widespread use of primaquine is limited by genetic factors, that may sometime found in > 40% of the population [80, 81].

The strategy to eliminate *P. vivax* is similar to that for *P. falciparum*, which includes tackling vectors and using chemoprevention, but fruitful elimination warrants additional interventions that take into account the complex nature of *P. vivax* infection. The available technology for detecting blood stages could emphasise on microscopic detection of low density *P. vivax* infections. Scope of control programmes needs to be enhanced to the level of detection of all *Plasmodium* species present in the blood of patient. It is paramount important to include G6PD test in malaria surveillance system to ensure better efficacy and safety of primaquine until an alternative safe and effective drug is available [82]. Field deployable sensitive and specific enough test to detect liver stage of *P. vivax* could be useful towards elimination of *P. vivax* malaria [83, 84].

### Imported cases – Analysis of problem

During the years 1955 to 2018, at least 30 countries and two territories were declared malaria free by the WHO. These nations are now facing the problem of imported malaria cases from other endemic countries. Increasing movement of people to and from endemic settings has largely connected the malaria free countries with malarious countries and therefore malaria pool remains the same [85–87]. Such situation continuously poses the serious threat in the detection and subsequent treatment as infrequent cases commonly remain unattended, particularly in non-malarious regions. Nevertheless, it is imperative that uninterrupted malaria importation into the malaria free and eliminating countries cause difficulty of diagnosis and restricting the spread of resistant parasites due to local transmission [88]. Even if the pay-off of un-accounted imported cases on malaria eliminating country is devastating, the data on malaria movement can be valuable to get information about malaria formula in the endemic countries and also to map down the malaria movement globally [87].

The importation of cases into non-endemic regions is governed by many factors, including endemicity and prevailing interventions in the region of origin. In addition, number of people travelling to endemic regions and frequency of travels also play major role in malaria importation. Since many decades, WHO AFR has been epicentre for exporting malaria cases into the non-endemic countries of Americas, European and other WHO regions [85, 86, 88–90]. Study has suggested that France and Britain suffer the highest number of imported malaria cases, mainly from WHO AFR [87, 91, 92]. The people of African ethnicity, frequently travelling to their country of origin in sub-Saharan Africa remain most affected mostly without suitable chemoprophylaxis [91]. Importation is also increasing with expanding business, historical and cultural ties between malaria eliminating countries and endemic countries [87]. During the past few years *P. falciparum* malaria import from African countries to China has increased due to Chinese investments and rising travel to these countries. Lai et al. [90] has conducted a systematic study to assess the driving factors involved in *P. falciparum* malaria importation from Africa to China and associated mortality. During 2011 to 2015, approximately 8653 *P. falciparum* cases (leading to 98 deaths) were imported from sub-Saharan countries into China. Most cases (91.3%) occurred in labour-related Chinese travellers to these countries. This study identified four strongly connected groups of African origin, and further reported that the number of imported cases was associated with the volume of air travellers to China, malaria parasite prevalence in Africa, and the amount of official involvement of China with investment in resource. The risk factors associated with the deaths from imported malaria cases corresponded to malaria diagnosis and different socioeconomic factors.

**Fig. 8:**
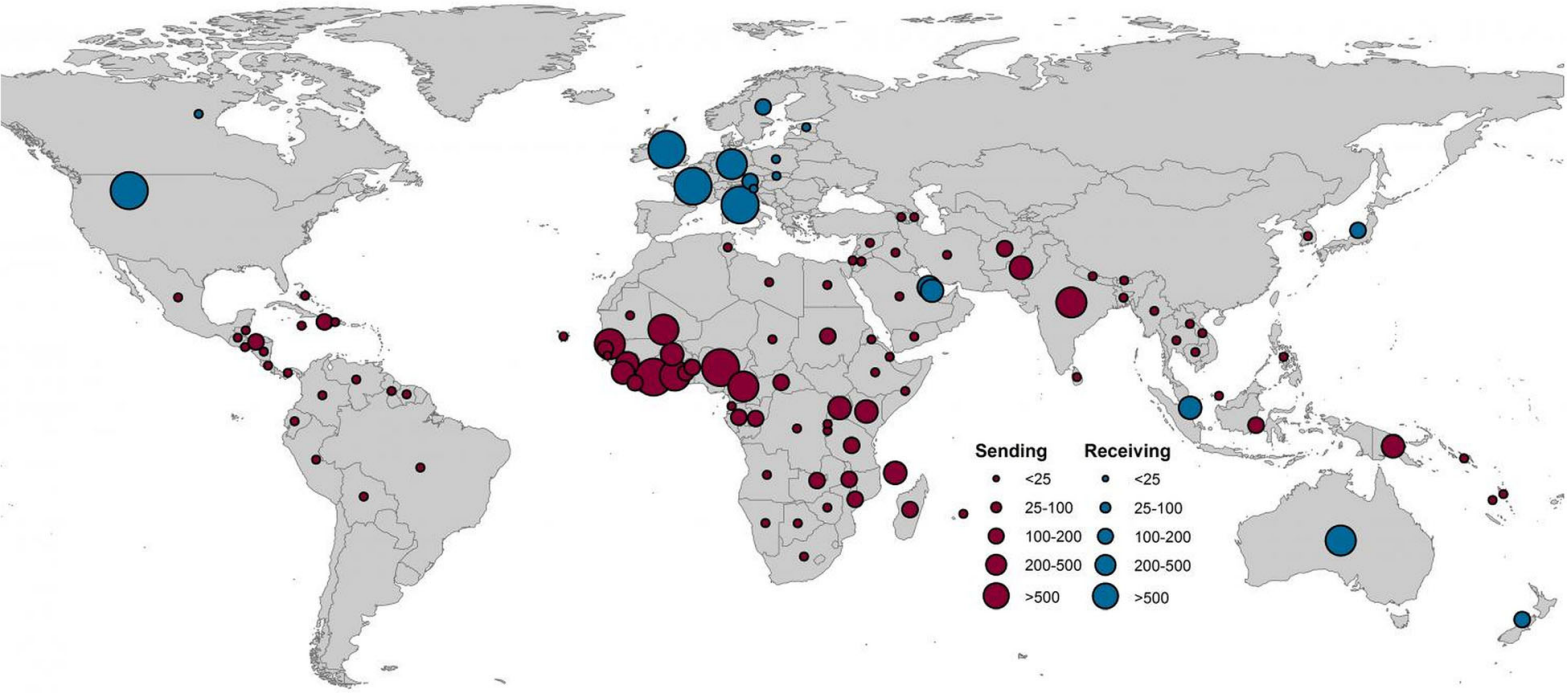
Estimated annual malaria import and export: Average annual malaria cases exported from endemic to non-endemic countries (shown in red) and imported to non-endemic countries from endemic countries (blue) (Figure taken from: Tatem et al., 2017)

Tatem et al. [87] has presented an analysis on the database of publicly reported statistics of imported malaria from 40 non-endemic countries, covering about 50 000 cases, for the past 10 years. They used the data to record the geographical variations in the importation of malaria cases into the malaria free or malaria eliminating countries. This study interestingly found that movement of malaria infected people followed specific routes during 2005 to 2015. The quantum of exported cases was very high in Africa and India, as more than 69% of such cases were traced exported from Africa and about 20% from India. Of those imported cases, majority of cases were recorded from France, United Kingdom, USA, Italy and Germany as compared to the other countries. Average annual cases imported by non-endemic countries from the endemic countries and exported by endemic countries to non-endemic countries have been depicted in Fig. 8.

Tatem and Smith [36] mapped probable source and destination of imported malaria cases and analysed census-based migration data to map communities in different countries that were linked by high levels of malaria infection movements. Study suggested that certain communities from West Africa and Central Asia were highly linked to malaria infection movement. Hence natural movement boundaries that govern the movement of infection between the regions and countries could be used to re-design malaria control programmes. Such strategy may differ from that adopted for more isolated countries. Another study [24] reported that in Bhutan, a country embarking on malaria elimination, more than 75% cases are recorded from three districts that are close to neighboring India, indicating malaria parasite import from India. Therefore malaria free countries such as Sri Lanka, that have high receptivity towards malaria infections and transmission due to the presence of several efficient vectors may find difficulty in tackling the imported malaria [93]. Several times it remains undetected because of low parasitaemia and non-specific initial symptoms including fever and headache among the patients.

Imported malaria challenges the elimination programmes in countries that are heading towards WHO malaria free certification. Although general health services in many countries undertake continuous vigilance of imported cases and provide prompt diagnosis and treatment, however surveillance mechanism is still weak in many endemic countries due to one or another reasons [1, 87, 89]. To counter this problem, in addition to strengthening the surveillance system, it is crucial to analyse country specific movement pattern of malaria cases. There is need to collaborate and strengthen control interventions with nations that export malaria cases regularly. Cross-border collaboration among the neighbouring countries, synchronising control efforts at regional level strict vigilance in highly connected locations could be most effective approach.

## Progress achieved towards malaria elimination

After about a decade of the launch of Roll Back Malaria programme in 1998 and Millennium Development Goals in the year 2000, malaria burden decreased globally [6]. This decline was largely escalated by development and scale up of effective intervention technologies, and encouraged by growing financial donorships and commitments at different levels in the endemic countries. Inspired by this steady achievement, it was called in for ‘malaria eradication’ in 2007 [94] and later supported by the WHO and various organisations workings on malaria elimination at global level [24–26, 95]. Endemic countries began re-defining control priorities and re-set aggressive elimination goals towards achieving this ambitious commitment. The concept of malaria elimination was better described from logistic, operational and epidemiological perspectives in countries that have relatively low malaria transmission [35, 36, 96–99].

**Fig. 9:**
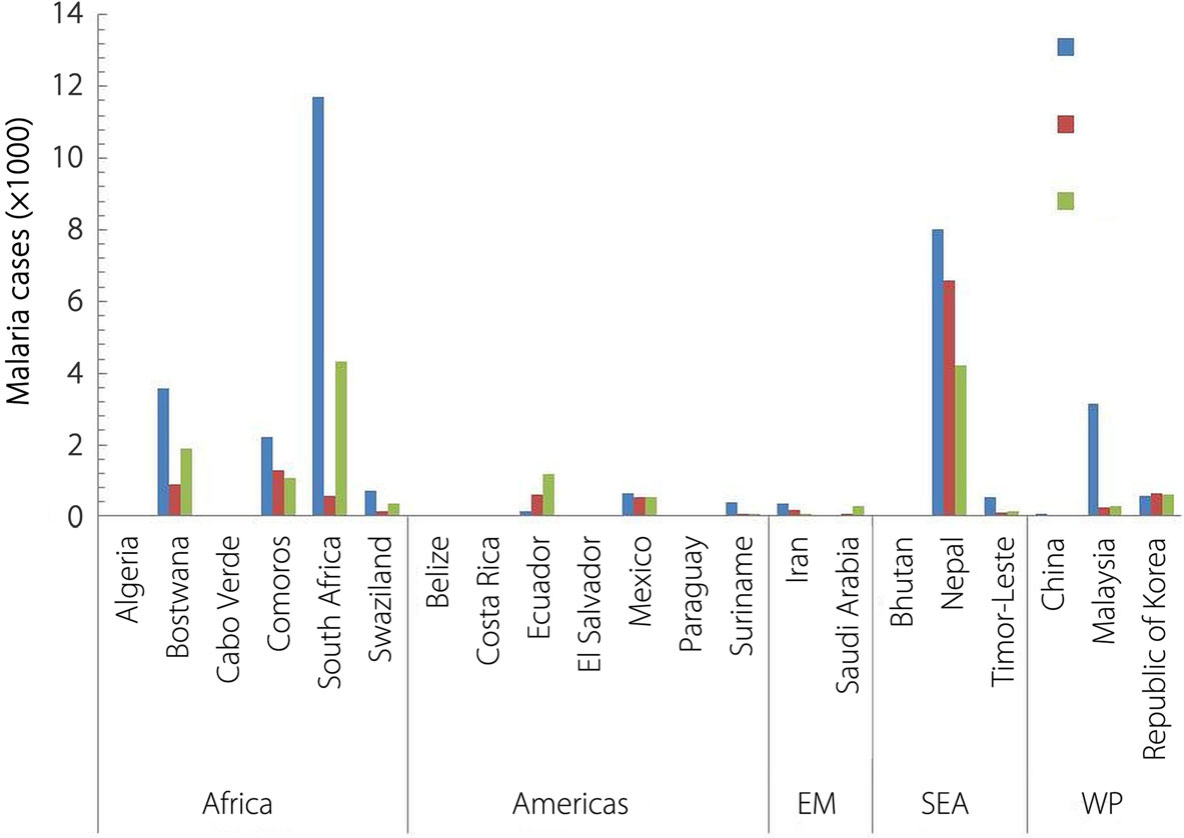
Malaria incidences in E-2020 nations: Malaria cases (× 1000) during 2014–2016 in the WHO assessed malaria eliminating countries (E-2020 countries; assessed to eliminate malaria by 2020) of WHO Regions. (EM: Eastern Mediterranean; SEA: South-East Asia; WP: Western Pacific). (Data taken from WHO Malaria Report, 2017)

In the year 2015, altogether 35 endemic nations met the WHO malaria-eliminating countries criteria. During the past two decades these nations have shown > 85% decline in malaria mortality and about 90% reduction in overall reported malaria cases [48, 100, 101]. Recently four countries namely, Kyrgyzstan, Maldives, Sri Lanka and Paraguay were declared malaria free after achieving zero malaria transmission at local level and fulfilling WHO criteria required for malaria free certification. Kyrgyzstan has become 19th countries in WHO ER to eliminate malaria, therefore interrupting the chain of malaria transmission into the region consisting of 53 countries. Maldives was declared malaria free in 2015 to successfully attain the status of first malaria free country in the WHO SEAR. Recently Sri Lanka was also certified malaria free after no reported local transmission during the past three years. Sri Lanka was among the most malaria endemic countries some decades ago, however it achieved the status before neighbouring India, Thailand, Bangladesh and other endemic countries in the region [102]. In WHO AR, with no record of malaria during the past five years, Paraguay became the first country in the past 45 years to have expunged malaria. Observing the malaria trends in endemic countries, WHO has aimed at wiping out malaria from 10 more countries by 2020 [1, 103] and estimated that 21 more countries (E-2020 countries), including six from WHO AFR, has potential to zero down the transmission in the coming years (Fig. 9).

This assessment has not been based merely on reported cases but also on the focused malaria objectives that include adequate improved intervention methods along with strong commitment [1, 9, 10]. Among these countries Paraguay has already been declared malaria free recently, while Algeria from WHO AFR has sustained zero malaria transmission for the last three years, therefore qualified for the malaria free certification. Costa Rica in WHO AR has also shown excellent achievement in recording no indigenous cases in 2014 and 2015, howbeit 4 cases were reported in 2016. China in the WHO WP region has displayed great consistency in reducing cases down to 3 in 2016 from 39 in 2015 and 6 in 2014 (Fig. 9).

Despite encouraging achievements, the malaria eliminating nations are facing substantial challenges in keeping with the declining cases. Many of the countries heading towards eliminations have not been able to maintain the ongoing intervention programmes, thereby leading to the upsurge of incidences [35, 98, 99, 104]. In initial phase of elimination efforts, the interventions including financial commitments are more focused and sustained towards aggressive control, however the fidelity of such programmes starts weakening once the cases are near to zero down. As a result countries start experiencing fresh spikes in malaria incidences. At least 13 countries (four each from WHO AFR and AR) that are in the WHO list of E-2020 countries did not display consistent decline in cases during the past three years (Fig. 9). Shretta et al. [105] has suggested that funds needed to eliminate malaria at initial level may be equal or more than that needed to control, but for sustained elimination, the cost is likely to be higher than that required for control.

Presently, the progress towards achieving malaria elimination goals has been contextualized by taking into account the country specific key factors such as surveillance, coverage status of key commodities, funding for malaria control and conflicts. Reports suggest that overall coverage with RDTs and ACTs is compromised during 2014–2016 and more than half of the countries with ongoing transmission were not on tract to reach targets of malaria reduction [1, 50]. Increased malaria burden between the years 2014 and 2016 indicates that efforts are not within the ambit of perceived targets [2, 7, 9, 10, 17, 50]. Taking into consideration the conservative estimates, that suggest global malaria case rise by about 5 millions in 2016 as compared to 2015, it has been apprehended that malaria burden is underestimated [1]. During the recent few years many endemic countries has reported decline in fund that were invested in malaria control and subsequently resulted in increase in malaria burden [1, 17, 28].

The overall gain in elimination during the past years has further strengthened the objective to attain complete malaria elimination. The global support in this context needs to be increased beyond the borders. However understanding of success achieved and experience earned needs to be disseminated to motivate the continuing efforts in nations heading towards elimination. Malaria elimination at global level requires collaborations among the malarious and malaria free countries along with their national level framework to restrict imported cases. Elimination efforts must be accelerated in high malaria burden countries that share most mortality and morbidity, however under no circumstances the malaria free or eliminating countries be ignored for the sustained malaria interventions. Most importantly, insufficient fund and resources in majority of high endemic countries still remains the major threat to the gains made so far towards achieving goals set in malaria elimination.

## Explicated role of national malaria control agencies

National programmes are backbone of elimination at country level and operate under diverse ecological, geo-political and financial circumstances. However well-defined region specific objectives are pre-requisite to attain goals in malaria control, elimination and eventual re-introduction. Control programmes at national level are committing to eliminate malaria on the call of WHO and other global agencies and considering to use proposition of different action frameworks including Global Technical Strategy [50, 106], President’s Malaria Initiative Strategy [107] and Action and Investment to Defeat Malaria by Roll Back Malaria [7], while simultaneously considering the region specific factors that may influence progress towards achieving goals. It is more likely that the control programmes set their objectives towards eliminating malaria at national level and at the same time to address the long term goal of global elimination. However there are challenges such as, poor health infrastructure, lack of trained staff and remote locations, which tend to weaken the progress of programmes regardless of concerted efforts. Therefore WHO has stressed upon ensuring commitment at all levels and strengthening health facility, in addition to addressing insecticide and antimalarial resistance [1, 50].

The control programmes are mostly integrated with the system of general health services, hence proper execution of interventions and maintaining quality becomes difficult [108]. Another serious drawback in the control programmes is lack of accountability, which in absence of sustained capacity building and strong supervision negates the control efforts. Sometimes decline in number of cases lowers the priority of programmes to continue interventions with same momentum. Such waning of control efforts has caused sudden rise in malaria incidences in several countries. In Turkey, irrigation projects led to increase in malaria transmission and subsequent outbreaks in 1977 [108, 109]. The control programmes in Turkey could not foresee the impact of such development on malaria incidences and did not sufficiently plan to control such inclined transmission. Similarly, outbreaks occurred in Cape Verde after operational delay and reduction in the use of interventions [108, 110, 111]. On the other hand, national programmes in many countries maintained consistent intervention activities even after achieving considerable reduction in the incidences. Another important responsibility of the malaria control agencies is to devise and adapt new strategies to keep up the pace of programmes. These may include rotational use of insecticides to deceive the resistance process and to increase entomological as well epidemiological surveillance in remote and border areas to diminish the transmission [112, 113].

At present, resurgence in incidences is more likely to threaten the demanding goal of global elimination. Fugitive inclines in the number of cases in countries that are eliminating malaria could be impeded by strong techno-financial commitments, consistency in implementation and adapting region specific flexibility in control programmes. The role of control programmes is not limited only to follow set guidelines but also to develop and ensure timely implementation of region specific strategies based upon in-depth surveillances to sustain elimination and prevent re-introduction. Furthermore programme agencies need to ascertain delivery of control services upto lowest possible level while fixing the responsibility of control staff at each level.

## Research focus and innovation for elimination

Malaria control programmes have implemented many evidence based intervention tools to reduce and eventually eliminate malaria scourge. It is plausible that the chosen tools have been well researched and found effective in the country of use. However more operational research is required to region specific intervention tools that could be relevant and scaled up to achieve maximum gain in elimination. Currently, the control programmes are less likely to be guided for dynamic heterogeneity in vector ecology, malaria transmission associated with the changing environment and socio-economical factors in the absence of parallel research on these aspects. Therefore progress using interventions may not be parallel to that expected. In spite of similar interventions followed at sub-national level, the operational limitations such as poor or underperforming health services, limited accessibility to health centres, socio-religious hindrances and deprived availability of sufficient protective measures to curtail transmission, undermine the efforts [14, 113–116]. Since each country has different set of challenges, the planning and implementation must be rationally based on research on prevailing epidemiology and entomology of malaria. Focus should be on careful selection of interventions that could address the specific requirements of the region [117, 118]. Implementation research could be an important approach to assess the effectiveness of intervention measures and to identify the gaps in malaria control. Region specific knowledge on vector mosquitoes, parasite species and other relevant factors that could influence the control interventions may guide changes needed to be incorporated in programmes for achieving desired success. Some recent studies suggest that research is not on priority of National Malaria Control Programmes globally and have very limited involvement in malaria operational research [117, 119, 120].

Bill and Melinda Gates Foundation reiteratedly emphasized surveillance as the backbone of effective malaria elimination. However at present sustained global elimination may not solely hinge on surveillance but require more explicit and quantitative tools. Malaria surveillance needs transformed in a way that could accelerate regional and national elimination efforts. Reducing the time for diagnosis and real time data harmonization for quick and precised interventions must be primary focus of current research. Efforts to increase accessibility of health facilities for obtaining recommended medicines and personal protection measures are warranted. New and more effective insecticides with different mode of action on mosquitoes could be useful in curbing the problem of insecticide resistance. At present research is more focused on developing insecticide resistance breaking compounds and improved surveillance tools that could be deployed in the field. However, it is equally pertinent to understand the malaria transmission capacity of resistant mosquitoes. Insecticide resistance could have impact on vectorial capacity of vectors and play crucial role in determining its longevity and vector competence to transmit the parasite. It is reported that resistance may reduce the ability of vectors to transmit disease, consequently overall epidemiological impact of resistance may not be much detrimental. In such a case, current focus on allocating considerable resources on insecticide resistance management might not be judicious [121]. On the other hand, insecticide resistance could improve the fitness and vectorial capacity of mosquitoes, hence warrants urgent need for effective resistance management [32, 121, 122]. Either way, the need is to understand the resistance evolution in vectors and merits priority research.

Additionally research on stratification plans of disease using more precised geographical information system and remote sensing may be encouraged and developed for focused allocation of resources in confirmed transmission areas [123–125]. A most advanced injectable vaccine candidate RTS, S (Mosquirix) been licensed for use, but has relatively little efficacy, hence apparently may not be sufficient enough singly to meet the objective of malaria elimination [126–128].

## Conclusions

Global malaria morbidity and mortality rates in the past two decades have asserted that the efforts are largely concerted, and slowly but steadily heading towards the common goal of malaria free world. However a brief incline in malaria cases during 2016 has raised fresh perturbation on whether elimination could be achieved on time or not [112]. For the countries that have eliminated malaria, it might be less desirable to maintain the pace of intervention efforts due to various constraints as compared to the high burden nations. Nevertheless maintaining zero malaria transmission and check on imported cases in malaria free countries, and further speeding up of interventions to clear transmission in elimination countries is most desirable, while discussing malaria trends globally. Strong collaboration backed by adequate political and financial support between the countries with a common agenda to eliminate malaria must be on priority. It is more important that funding be more focused on effective and timely imposition of control intervention in endemic and hard to reach regions.

Complete elimination is not possible until population particularly that inhabiting the low endemic settings are not sensitized. Success tales of previously endemic countries such as Sri Lanka and more recently Paraguay demonstrate that elimination is possible in every endemic country. The policy makers at regional levels need to take lessons from setbacks and reshape control strategy accordingly. Majority of control programmes, even today, are planned without taking into account the participatory activity of people living in endemic areas and implemented without considering that success of programmes is limited by receptive behaviour of people. Therefore promoting knowledge through campaigns could be crucial to enhance intervention receptivity. Furthermore, strategy to address other malaria parasites that cause infrequent infection in human is needed to be evolved and implemented. Developing guidelines on parasite level for categorizing a patient as asymptomatic and diagnostic procedures targeting asymptomatic carriers could be important while aiming at complete elimination. Additionally, adequate health infrastructure, accurate knowledge on prevailing vectors, socio-political commitment, deploying region specific intervention strategies and involving local communities into malaria report, research and elimination would be imperative in complete malaria elimination.

## Additional files

Additional file 1: Multilingual abstracts in the five official working languages of the United Nations. (PDF 456 kb)

Additional file 2: **Table S1.** Treatment failure rate (median) for *P. falciparum* in malaria endemic countries on the basis of studies after the year 2010. (XLS 71 kb)

Additional file 3: **Table S2.** Treatment failure rate (median) for *P. vivax* in malaria endemic countries on the basis of studies after the year 2010. (XLS 60 kb)

## Abbreviations

ACT: Artemisinin-based combination therapy
AFR: African Region
AR: Americas Region
CYP2D6: Cyt-P450 polymorphism
EMR: Eastern Mediterranean Region
ER: European Region
G6PD: Glucose-6-phosphate dehydrogenase
IRS: Indoor residual spray
ITNs: Insecticide-treated nets
LLINs: Long-lasting insecticide-treated nets
RDTs: Rapid diagnostic tests
SEAR: South-East Asian Region
WHO: World Health Organisation
WPR: Western Pacific Region
