# Correction to: Are malaria elimination efforts on right track? An analysis of gains achieved and challenges ahead

**DOI:** 10.1186/s40249-019-0527-7

**Published:** 2019-03-16

**Authors:** Sunil Dhiman

**Affiliations:** Vector Management Division, Defence Research and Development Establishment, Gwalior, Madhya Pradesh 474002 India

**Correction to: Infect Dis Poverty**

**https://doi.org/10.1186/s40249-019-0524-x**

In the original publication of this article [1], the legends in Fig. 9 are missing during the process of typesetting. The correct Fig. 9 should be as below. The original publication has been corrected.

**Fig. 9:**
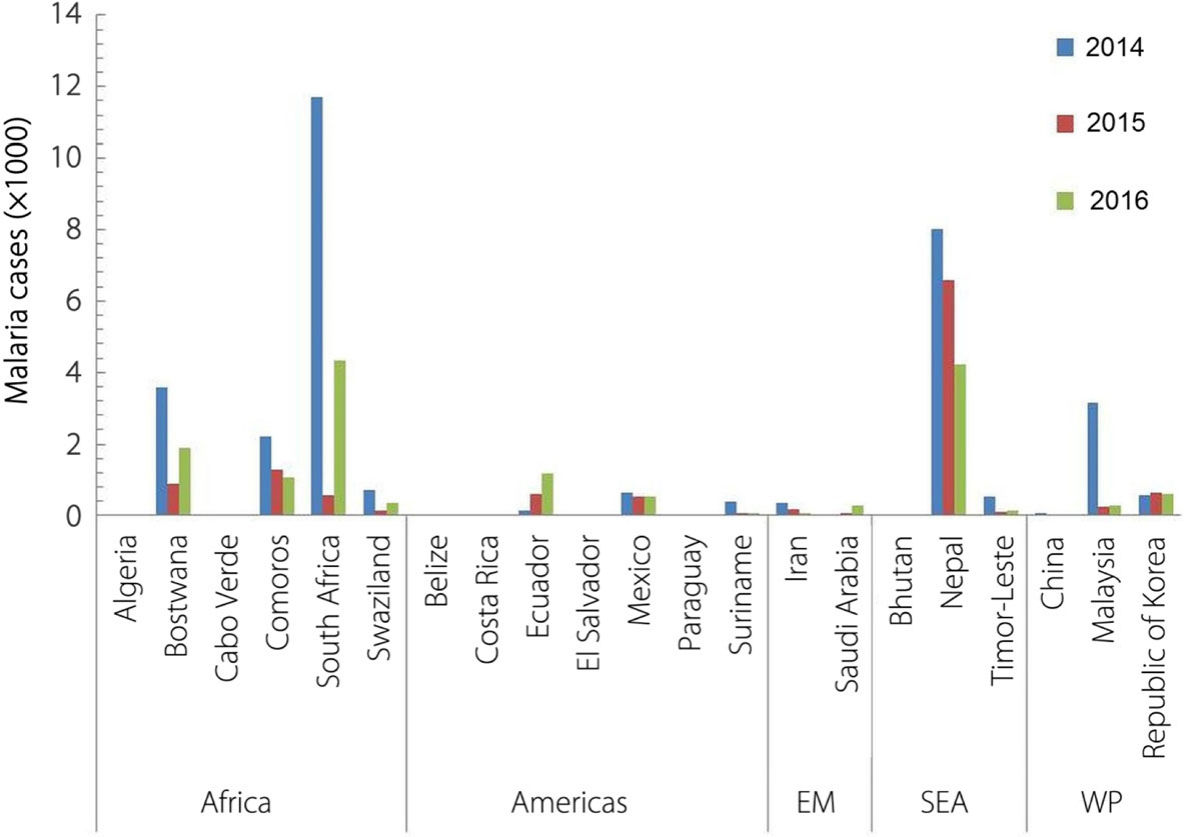
Malaria incidences in E-2020 nations: Malaria cases (× 1000) during 2014–2016 in the WHO assessed malaria eliminating countries (E-2020 countries; assessed to eliminate malaria by 2020) of WHO Regions. (EM: Eastern Mediterranean; SEA: South-East Asia; WP: Western Pacific). (Data taken from WHO Malaria Report, 2017)
