# Supplementary material for: Are malaria elimination efforts on right track? An analysis of gains achieved and challenges ahead
Source: Infect Dis Poverty. 2019 Feb 13;8:14. doi: 10.1186/s40249-019-0524-x (PMC6375178; doi:10.1186/s40249-019-0524-x)
Supplement: Supplementary file 1 — Multilingual abstracts in the five official working languages of the United Nations. (PDF 456 kb) [file 40249_2019_524_MOESM1_ESM.pdf]

Translation of the abstract into the five official working languages of the United Nations

هل تسير جهود القضاء على الملاريا في الطريق الصحيح؟ تحليل للمكاسب التي تحققت والتحديات المقبلة

سونيل ضيمان

ملخّص

الخلفية: تسبب الملاريا معدلات مرضية ووفيات كبيرة كل عام. في السنوات القليلة الماضية، انخفضت حالات الملاريا في العالم، وتتجه البلدان الموبوءة بالمرض نحو القضاء عليه. ومع ذلك، يبدو أن خفض عدد حالات الملاريا أسهل من القضاء المستمر على المرض. ولأجل تحقيق هدف التخلص الكامل من الملاريا والمحافظة على حالة القضاء على المرض، فمن الضروري تقييم المكاسب التي تحققت خلال السنوات الأخيرة.

النص الرئيسي: مع وجود الدعم العالمي وجهود منظمة الصحة العالمية التي تصب في هذا الاتجاه، تم تنفيذ برامج المكافحة بشكل فعال في العديد من البلدان الموبوءة. وبالنظر إلى الاهتمام المثار حول الموضوع والاستثمارات في برامج القضاء على الملاريا على المستوى العالمي، يبدو أن الهدف الطموح للقضاء على المرض ممكن. وقد لعبت التدخلات المستدامة دوراً محورياً في تقلص الملاريا، إلا أن مقاومة الأدوية ومبيدات الحشرات، والمعتقدات والممارسات الاجتماعية والديموغرافية والثقافية والسلوكية، والبنية التحتية الصحية - التي تحتاج الى قدر عظيم من الاصلاح - يمكن أن تحرف التقدم الذي تحقق حتى الآن عن مساره. إن تجاهل مثل هذه العوامل المعوقة المقرونة بعوامل معينة - خاصة بالمنطقة - قد يعرض للخطر قدرتنا على الالتزام بالمسار الصحيح لبلوغ هدفنا المتمثل بالقضاء على طفيل الملاريا على المستوى العالمي. على الرغم من أهمية الدعم خارج المناطق، إلا أن النهج المتكامل المُدار بشكل جيد لمكافحة ناقلات الأمراض على المستويين الإقليمي والقطري باستخدام تدخلات خاصة بالمنطقة - مختارة بدقة - تستهدف كلاً من الناقل والطفيل إلى جانب مشاركة المجتمع هو أمر ضروري. وقد أدى انحراف سريع في مسار مكافحة الملاريا خلال عام 2016 إلى إثارة اضطراب جديد حول ما إذا كان بالإمكان تحقيق القضاء على المرض في الوقت المحدد أم لا.

الاستنتاجات: يمكن لأدوات التدخل المتاحة حالياً أن تقلل على الأرجح من فرص انتقال المرض، لكن إزالة المراكز السطحية للملاريا من المناطق - التي يمكن أن يتسع فيها نطاق المرض في أي وقت - غير ممكن من دون إشراك السكان المحليين. وبالرغم من ذلك، فإن استمرارية عدم انتقال الملاريا وفحص المستوردات (لضمان خلوها من المرض) في الدول المعلن خلوها من الملاريا والإسراع أيضاً في التدخلات التي تهدف لوقف انتقال المرض في البلدان التي تحاول القضاء عليه هي أمور مرغوبة للغاية. إن التعاون القوي المعزز بدعم سياسي ومالي كافيين فيما بين البلدان التي يجمعها هدف مشترك للقضاء على الملاريا يجب أن يكون على رأس الأولويات. تسعى المراجعة الحالية إلى تقييم التقدم المحرز في القضاء على الملاريا خلال السنوات القليلة الماضية، وتسلط الضوء على بعض القضايا التي يمكن أن تكون مهمة في القضاء على الملاريا بنجاح.

Translated from English version into Arabic by Ammar Alhosain, proofread by Amal Imam, through

TRANSLATORS WITHOUT BORDERS

疟疾消除工作是否在正确的轨道上？——分析取得的成果和面临的挑战

Sunil Dhiman

摘要

引言：每年疟疾的发病率和死亡率都很高。在过去几年中，全球疟疾病例正在减少，流行国家正在逐步消除疟疾。为了实现彻底消除和保持消除状态这一目标，有必要对近年来取得的成果进行评估。

主要内容：在全球支持和世界卫生组织(WHO)的努力下，防治规划已在许多流行国家得到有效实施。鉴于疟疾消除计划已引起了全球关注和投资，消除疟疾这一宏伟目标看来是可行的。可持续的干预措施在疟疾病例减少方面发挥了关键作用，但是药物和杀虫剂的抗药性、社会、人口、文化和信仰，以及未经改造的卫生基础设施等因素可能会使已取得的进展发生倒退。忽视这些阻碍因素，再加上某些特定的区域因素，可能会使我们偏离正常轨迹，影响我们实现全球消除疟疾的目标。虽然在地区之外提供支持很重要，但必须在区域和国家一级采用管理良好的综合病媒防制办法，谨慎选择针对病媒和寄生虫以及社区参与的特定区域干预措施。2016 年疟疾疫情的短暂上升使人们对是否能够及时消除疟疾产生了新的不安。

结论：目前可用的干预工具能最大限度地减少传播，但如果不涉及当地人群，就不可能从疟疾暴发地区消除疟疾。在已宣布零疟疾病例的国家保持零疟疾传播，对输入性疟疾病例进行检查，并进一步加快干预措施以阻止疟疾在消除国家传播是最可取的。在以消除疟疾为共同目标的国家之间获得充分的政治和财政支持进行强有力合作是当务之急。本文评估了过去几年消除疟疾取得的进展，并提出可能影响消除疟疾的几个重要问题。

Translated from English version into Chinese by Hao-Nan Wu, edited by Pin Yang

CHINESE CENTER FOR DISEASE CONTROL AND PREVENTION
NATIONAL INSTITUTE OF PARASITIC DISEASES

Les efforts visant à l’éradication du paludisme sont-ils sur la bonne voie ? Une analyse des acquis et des défis à venir

Sunil Dhiman

Résumé

Contexte: Le paludisme entraîne une morbidité et une mortalité élevées chaque année. Au cours des années qui se sont écoulées, le nombre de cas de paludisme diminue et les pays endémiques progressent sur la voie de son éradication. Néanmoins, la réduction du nombre de cas semble être plus aisée que d’assurer une éradication pérenne. La satisfaction de l’objectif d’éradication intégrale et le maintien de cet état nécessitent une évaluation des acquis obtenus au cours des dernières années.

Texte principal: Grâce au soutien international et aux efforts de l’Organisation Mondiale de la Santé (OMS), les programmes de lutte ont été mis en œuvre de manière efficace dans de nombreux pays endémiques. Au vu de l’intérêt éveillé et des investissements réalisés en faveur de programmes d’éradication du paludisme à l’échelle internationale, l’objectif ambitieux que constitue l’éradication semble faisable. Des interventions pérennes ont joué un rôle clé dans la contraction du paludisme, mais la résistance aux médicaments et aux insecticides, les croyances et les pratiques sociales, démographiques, culturelles et comportementales, ainsi qu’une infrastructure sanitaire non réformée risquent de mettre à mal les acquis obtenus jusqu’à présent. Le fait d’ignorer de tels obstacles associé à certains facteurs spécifiques régionaux sont susceptibles de porter préjudice à notre capacité de poursuivre les efforts visant à éradiquer le parasite du paludisme dans le monde entier. Bien que

l’assistance au-delà des territoires soit importante, une approche pertinente de gestion intégrée des vecteurs aux échelles régionale et nationale sur la base d’interventions spécifiques à une zone donnée ayant été scrupuleusement choisies ciblant à la fois le vecteur et le parasite en plus de l’intégration de la communauté est nécessaire. Une brève tendance à la baisse en 2016 a récemment perturbé les acteurs sur la possibilité ou non d’éradiquer cette maladie en temps voulu.

Conclusions: Les outils d’intervention actuellement disponibles peuvent très probablement réduire la transmission de la maladie, mais l’élimination des épicentres du paludisme, à partir desquels la maladie peut de nouveau se propager, n’est pas possible sans intégrer la population locale. Le maintien d’un taux zéro de transmission du paludisme et les contrôles sur l’importation du paludisme dans des pays déclarés exempts de cas de paludisme, ainsi que la poursuite de l’accélération des interventions visant à stopper la transmission dans les pays où la maladie doit encore être éradiquée sont néanmoins plus que souhaitables. Une forte collaboration appuyée par un soutien politique et financier approprié parmi les pays poursuivant le même but, l’éradication du paludisme, doit revêtir une absolue priorité. La présente révision doit permettre d’évaluer les acquis obtenus dans le cadre de l’éradication du paludisme au cours des dernières années et mettre en valeur certains aspects qui pourraient s’avérer importants pour la réussite des efforts d’éradication.

Translated from English version into French by Eric Ragu, proofread by Latifa El Machichi, through

TRANSLATORS
WITHOUT BORDERS

Оправданы ли усилия по борьбе с малярией? Анализ достигнутых успехов и возможных сложностей

Сунил Дхиман

Аннотация

Предыстория: Ежегодно малярия является причиной высокой заболеваемости и смертности. За последние несколько лет в мире наблюдается сокращение количества случаев заболевания малярией, а эндемичные страны движутся в сторону ликвидации малярии. Тем не менее, добиться сокращения количества случаев заболевания кажется более простой задачей, чем полная ликвидация малярии. Поэтому, для полной ликвидации болезни и поддержания этого статуса, необходимо проанализировать достижения последних лет.

Основной текст: С растущей поддержкой мирового сообщества, усилиями Всемирной Организации Здравоохранения (ВОЗ) во многих странах, эндемичных по малярии, были введены программы контроля. С учетом высокого интереса и инвестиций в программы по ликвидации малярии на мировом уровне, амбициозная задача искоренения этой болезни представляется решаемой. Последовательные меры сыграли решающую роль в сокращении количества случаев заболевания малярией, однако устойчивость к лекарственным препаратам и инсектицидам, социальные, демографические, культурные и личные убеждения и практики, а так же не

прошедшая реформу инфраструктура здравоохранения могут свести на нет достигнутый прогресс. Игнорирование этих препятствующих факторов, а также специфических сложностей на уровне региона, могут поставить под угрозу способность оставаться на верном пути к полной ликвидации возбудителей малярии в мире. Несмотря на важность поддержки извне, необходим слаженный комплексный подход к контролю численности переносчиков заболевания на региональном и национальном уровне, с вовлечением местного населения, используя тщательно подобранные меры, зависящие от конкретной местности и направленные как на переносчика, так и на паразита. Кратковременное увеличение числа случаев малярии в 2016 году вызвало новую волну беспокойства о возможности искоренения малярии.

Выводы: Средства борьбы с болезнью, доступные в настоящее время, вероятнее всего способны замедлить ее распространение. Однако, устранение очагов малярии, где заболевание может вспыхнуть в любой момент, не представляется возможным без вовлечения местного населения. Тем не менее, крайне желательны сохранение нулевого роста числа случаев передачи болезни, предотвращение импорта малярии в страны, свободные от малярии, а также принятие ускоренных мер по остановке распространения болезни в странах, где ведется борьба с малярией. Тесное сотрудничество стран с общей задачей искоренения малярии, при необходимой политической и финансовой поддержке, должно быть приоритетной задачей. Данный обзор является попыткой оценить прогресс, достигнутый в борьбе с малярией за последние несколько лет. Он подчеркивает ряд проблем, решение которых может сыграть важную роль в успешной борьбе с малярией.

Translated from English version into Russian by Lilia Zaripova, proofread by Anna Krasnopeeva, through

TRANSLATORS WITHOUT BORDERS

¿Van por buen camino los esfuerzos por eliminar el paludismo? Un análisis de los logros alcanzados y de los retos que hay por delante

Sunil Dhiman

Resumen

Antecedentes: El paludismo causa una morbilidad y una mortalidad significativas cada año. En los últimos años, los casos mundiales de paludismo están disminuyendo y los países endémicos se encaminan hacia su eliminación. Sin embargo, reducir el número de casos parece ser más fácil que la eliminación permanente. Por lo tanto, para lograr el objetivo de la eliminación completa y mantener la situación de eliminación, es necesario evaluar los avances logrados en los últimos años.

Texto principal: Gracias al apoyo mundial y a los esfuerzos de la Organización Mundial de la Salud (OMS), los programas de control se han aplicado eficazmente en muchos países endémicos. Dado el interés suscitado y las inversiones en programas de eliminación del paludismo a nivel mundial, el ambicioso objetivo de la eliminación parece factible. Las intervenciones sostenibles han desempeñado

un papel fundamental en la reducción del paludismo, pero la resistencia a los medicamentos y los insecticidas, las creencias y prácticas sociales, demográficas, culturales y de comportamiento, y la falta de reformas en la infraestructura sanitaria podrán hacer retroceder los progresos logrados hasta la fecha. Ignorar estos factores limitantes, junto con ciertos factores específicos de la región, puede poner en peligro nuestra capacidad de seguir el camino correcto para lograr la eliminación mundial del parásito del paludismo. Aunque el apoyo más allá de los territorios es importante, es necesario un enfoque bien administrado de la gestión integrada del vector a nivel regional y nacional, utilizando intervenciones específicas en áreas seleccionadas escrupulosamente, dirigidas tanto a vectores como a parásitos, junto con la participación de la comunidad. Una breve inclinación en el paludismo durante 2016 ha provocado nuevas perturbaciones sobre si la eliminación podrá lograrse a tiempo o no.

Conclusiones: Es muy probable que las herramientas de intervención disponibles en la actualidad puedan reducir la transmisión, pero la eliminación de los epicentros del paludismo, desde los que la enfermedad puede brotar en cualquier momento, no es posible sin la participación de la población local. Sin embargo, lo más aconsejable es mantener la transmisión del paludismo en cero y controlar las importaciones de paludismo en los países declarados libres de paludismo, así como acelerar aún más las intervenciones para detener la transmisión en los países en vías de eliminación. Una colaboración sólida respaldada por un apoyo político y financiero adecuado entre los países con el objetivo común de eliminar el paludismo debe de ser la máxima prioridad. En el presente examen se intenta evaluar los progresos logrados en la eliminación del paludismo en los últimos años y se destacan algunas cuestiones que podrán ser importantes para el éxito en la eliminación del paludismo.

Translated from English version into Spanish by Lia Sarra Felip, proofread by Mayra León, through

TRANSLATORS
WITHOUT BORDERS
